# High Density Lipoprotein-Based Therapeutics: Novel Mechanism of Probucol in Foam Cells

**DOI:** 10.3389/fcvm.2022.895031

**Published:** 2022-04-26

**Authors:** Anouar Hafiane, Annalisa Ronca, Robert S. Kiss, Elda Favari

**Affiliations:** ^1^Research Institute of the McGill University Health Center, Montreal, QC, Canada; ^2^Department of Food and Drug, University of Parma, Parma, Italy

**Keywords:** probucol, cellular cholesterol efflux, atherosclerosis, foam cells/macrophages, HDL

Since the 1970's, high density lipoprotein (HDL) has been an active research topic due to epidemiological studies, such as the Framingham study and many others, that established the inverse association between HDL cholesterol (HDL-C) levels and the prevalence of coronary heart disease (CHD) (1). The deceptive results of HDL-based therapies (inhibitors of cholesteryl ester transfer protein, niacin, and apolipoprotein A-I (apoA-I) infusion therapies), and Mendelian randomization approaches do not fully support a causal association between HDL-C and cardiovascular protection (2). Thus, the role of HDL in health and disease is more complex than anticipated (3). Consequently, there has been a paradigm shift in the study of HDL as a therapeutic target, from the measurement of HDL concentration to the evaluation of HDL function (i.e., cholesterol efflux capacity) (4). Growing evidence proposes that cardiovascular morbid conditions alter the HDL composition and roles transforming it from healthy and functional into pro-atherogenic and dysfunctional (5). A key player in the HDL metabolic pathway that has been substantially explored by various agonists is the ATP-binding cassette transporter A1 (ABCA1) defined as the rate-limiting factor in the formation of HDL (6). This transporter mediates cellular cholesterol and phospholipid removal to generate nascent HDL (nHDL). The most extensively studied function of HDL is the ability to promote net cellular cholesterol efflux. However, the regulation of ABCA1 receptor expression is complex and poorly understood and the physiological and clinical relevance of such a treatment remains uncertain. In the current issue of BBA Advances, we report the findings of our cellular studies on a new mechanism in foam cell macrophages that is ABCA1-independent, and revealed through the use of probucol (7). Although clinical trials were stopped (8), probucol is still being investigated for its effect on the inhibition of atherosclerosis initiation *in vitro* and in animal models. Of interest, probucol trials still ongoing suggest potential benefits on CHD on top of conventional therapy (9). Basically, probucol is known to act as an ABCA1 inhibitor (10), although the method of addition of probucol to cells or animals may explain some of the differences observed in the inhibitory activity. We show that probucol treated THP-1 foam cells are still able to induce the release of cholesterol-containing small nHDL particles with a diameter of more than 7 nm in an ABCA1-independent manner. In support, we demonstrate that ABCA1 expression is the same in non-foam and foam cells, despite different efflux levels. Quantitative data show that probucol only partially inhibits the transfer of cholesterol into nHDL particles. Interestingly, the release of these probucol-nHDL were active in HDL biogenesis, supporting the contention that these particles are potentially atheroprotective, especially when macrophage-derived cholesterol is involved (Table 1). Indeed, the ABCA1-independent activity influencing the total accessible plasma membrane cholesterol level that remains in foam cells is consistent with the concept that lipids within nHDL originate from specific domains in the plasma membrane. A previous study by Yamamoto et al. demonstrated that probucol enhanced the release of cholesterol from foam cells but with no description of ABCA1's role (11, 14–16, 19). Despite a paradox surrounding the lipid lowering effect of probucol (Table 1), these findings align with data supporting the potential antiatherogenic role of probucol. Indeed, previous studies indicated positive effects of probucol on atherosclerosis treatments *in vitro* and *in vivo* (19, 24, 25), however some clinical data indicated negative effects of probucol (26). In addition, a role of probucol was observed in reducing micro-particles release mainly those rich in cholesterol with size range from various cell lines (50–250 nm) (Table 1) (18). Use of probucol unveiled a novel and specific pathway in foam cells where functional cholesterol efflux and formation of nHDL is enhanced in the absence of ABCA1 activity. This activity was not observed in non-foam cells. Moreover, probucol incorporation significantly influences lipid droplet morphology and size (7). This is relevant to lipids droplets roles in mammalian innate immunity, triglyceride synthesis, and mitochondrial dynamics (27, 28). These observations will clarify the mechanisms by which HDL can be protective especially in foam cells. However, the physiological and clinical importance of such approaches remains to elucidated, and substantial additional preclinical work will be required. Exploring new HDL generating pathways that enhance cholesterol efflux is a prospect of a completely novel strategy to raising plasma HDL concentration for CHD prevention that might succeed where other approaches have failed. However, because of the unsatisfactory track record of HDL-based therapies, further research is imperative before renewing our enthusiasm for HDL as a target for therapy. Despite the fact that we have not the ability of probucol to enhance an ABCA1-independent pathway, we suggest the possibility to use probucol as a tool to probe intracellular cholesterol trafficking to inhibit ABCA1. Overall, this may provide substantial evidence for a revised model of cholesterol trafficking in macrophages foam cells. In our opinion this is a new argument in HDL metabolism among cardiovascular researchers if probucol has clinical significance. There is compelling purposes to believe that this old controversial medication has much more to offer than previously known.

**Table 1:** Summary of potential mechanisms of pharmacologic action of probucol.

**Mechanism**	**Model**	**References**
Inhibits ABCA1 cholesterol efflux activity	THP-1 and J774 cells	(11, 12)
ABCA1-independent activity in foam cells	THP-1 cells	(7)
Modification in lipid droplets morphology		
Increases LDL catabolism independent of the LDL receptor	Human fibroblasts	(13)
Probucol-Oxidized Products: Spiroquinone and diphenoquinone	RAW264.7 cells	(14)
Inhibit binding of lipid free apoA-I to the cells	Human fibroblast WI-38 HepG2	(15–17)
Protects ABCA1 from calpain-mediated degradation	THP-1 cells	(11)
Decreases micro-particles containing cholesterol release (50–250 nm)	THP-1, BHK, HEPG2 cells	(18)
Reduces xanthomas and atheromatous vascular lesions	UE-12 and THP-1 cells Tendon xanthomas	(19)
Inhibits cellular cholesterol efflux in cells	J774 mouse macrophages	(12)
Increases HDL biogenesis	THP-1 cells	(11)
Prevents foam cell formation	THP-1 cells	(19)
Activates nHDL formation in foam cells	THP-1 cells	(7)
Selective reduction in HDL2 particles size of FH patients*	FH patient's plasma	(19)
Prevents the oxidative modification of LDL	Rabbit aortic endothelial cells	(20)
Increases plasma LCAT, CETP activities, and apoE concentration**	FH patient's plasma	(21)
Improve HDL function (anti-inflammatory and anti-oxidant)	New Zealand white rabbits	(22)

*ABCA1, ATP-binding cassette transporter A1; THP-1, Tamm–Horsfall protein 1; LDL, low density lipoprotein; nHDL, nascent high density lipoprotein; ApoA-I, apolipoprotein A-I; BHK, baby hamster kidney cells; LCAT, lecithin-cholesterol acyltransferase; CETP, cholesteryl ester transfer protein; FH, familial hypercholesterolemia*.

*Smaller HDL particles may be biologically more active and beneficial to the reverse cholesterol transport from peripheral tissue to the liver (19).

**This was viewed as consistent with a postulated increase in reverse cholesterol transport via the remnant pathway (23).

## Author Contributions

AH conceptualized, wrote, edited, and revised the manuscript. AR, RK, and EF edited and revised the manuscript. All authors approved the submitted version.

## Funding

AH received support by Postdoctoral Fellowships from the Canadian Institutes of Health Research (CIHR, RN408399 – 430975).

## Conflict of Interest

The authors declare that the research was conducted in the absence of any commercial or financial relationships that could be construed as a potential conflict of interest.

## Publisher's Note

All claims expressed in this article are solely those of the authors and do not necessarily represent those of their affiliated organizations, or those of the publisher, the editors and the reviewers. Any product that may be evaluated in this article, or claim that may be made by its manufacturer, is not guaranteed or endorsed by the publisher.
